# P-745. Skin and Soft-tissue Infections among Military Recruits

**DOI:** 10.1093/ofid/ofae631.941

**Published:** 2025-01-29

**Authors:** Katrin Mende, Matthew Igo, Ian Seibert-Parzyszek, M Leigh Carson, Julian Davies, Laveta Stewart, David Tribble

**Affiliations:** Infectious Disease Clincial Research Program, JBSA Ft Sam Houston, Texas; Infectious Disease Clinical Research Program, Bethesda, Maryland; Infectious Disease Clinical Research Program, Bethesda, Maryland; Infectious Disease Clinical Research Program, Department of Preventive Medicine and Biostatistics, Uniformed Services University of the Health Sciences, Bethesda, MD, USA, Bethesda, MD; Infectious Diseases Clinical Research Program, Henry M. Jackson Foundation, Bethesda, Maryland; Infectious Disease Clinical Research Program, Henry Jackson Foundation, Bethesda, Maryland; Infectious Disease Clinical Research Program, Department of Preventive Medicine and Biostatistics, Uniformed Services University of the Health Sciences, Bethesda, MD, USA, Bethesda, MD

## Abstract

**Background:**

Skin and soft-tissue infections (SSTIs), commonly *Staphylococcus aureus*, are an important cause of morbidity in congregate military populations with high rates among military recruits. We characterized SSTI epidemiology over an ∼10-year period at a military training facility in GA (largest U.S. training facility; ∼30,000 recruits per year).

**Figure:**
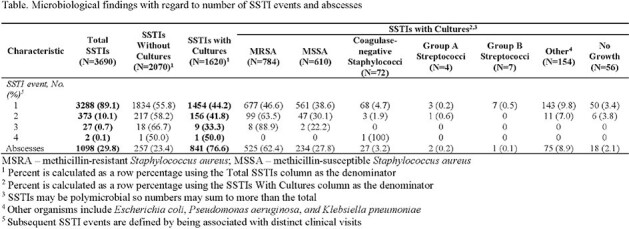

**Methods:**

SSTI data were collected from US Army Infantry recruits during basic training, through four studies (2 observational and 2 interventional studies spanning 2010-2019) and pooled into a single resource for retrospective research. SSTI characteristics (syndromes and diagnosis timing) and microbiology are described.

**Results:**

The study population includes 3288 recruits who developed SSTIs during the training period for a total of 3690 SSTIs. Median age was 19 years and median time for initial SSTI development was 7 (IQR 4 -11) weeks of training. Cellulitis (N=1600, 43%) and abscesses (N=1098, 30%) were most common and median time to diagnosis was 7 (IQR 4-11) and 8 (IQR 4-11) weeks of training, respectively. Cultures were collected from 1620 (44%) SSTIs, including 77% of abscesses. Methicillin-resistant and methicillin-susceptible *S. aureus* (MRSA and MSSA) were identified in 784 (48%) and 610 (38%) cultures, respectively (**Table**). Other findings include coagulase-negative staphylococci (4%), group A streptococci (0.2%), and group B streptococci (0.4%). A total of 373 recruits had ≥ 2 SSTIs and 55% of the subsequent 402 SSTIs were abscesses. Median time to 2^nd^ and 3^rd^ SSTIs was 10 (IQR 7.5-12) and 12 (IQR 10-13) weeks of training, respectively. There was a significant difference among the bacterial distribution for abscesses (p< 0.001) with MRSA contributing the highest proportion (62%), followed by MSSA (28%). MRSA was also identified with 64% of the 166 subsequent SSTIs with cultures.

**Conclusion:**

Cellulitis and abscesses are frequent infections affecting military recruits during high-intensity congregate training. The most common bacteria with purulent infections (i.e., abscesses), as well as subsequent infections, was MRSA. Pooling data from multiple studies allows for examination of syndromes and etiology to inform targeted interventions.

**Disclosures:**

**David Tribble, MD, DrPH**, AstraZeneca: The IDCRP and HJF were funded to conduct an unrelated phase III COVID-19 monoclonal antibody immunoprophylaxis trial as part of US Govt COVID Response

